# From Microbiota to Metabolomics: How *Corylus heterophylla* Fisch. Male Flower Extract Shields Mice from Cognitive Decline

**DOI:** 10.3390/nu17243958

**Published:** 2025-12-18

**Authors:** Wei Lu, Yujie Li, Xinyuan Liao, Han Hu, Bolin Zhang, Lisong Liang, Haina Gao

**Affiliations:** 1 School of Food and Health, Beijing Technology and Business University, Beijing 100048, China; 19979779160@163.com (W.L.); liyujie_2001@163.com (Y.L.); liaoxy202410@163.com (X.L.); 2 Bee Research Institute, Chinese Academy of Agricultural Sciences, Beijing 100093, China; wuhan_hh@126.com; 3 Beijing Key Laboratory of Forest Food Processing and Safety, Department of Food Science and Engineering, College of Biological Sciences and Technology, Beijing Forestry University, Beijing 100083, China; zhangbolin888@163.com; 4 Key Laboratory of Silviculture of State Forestry Administration, Research Institute of Forestry Chinese Academy of Forestry, Beijing 100091, China; lianglscaf@126.com

**Keywords:** cognitive decline, hippocampal neuroinflammation, *Corylus heterophylla* Fisch. male flower extract, microbiota-gut-brain axis, flavonoids, microglial polarization

## Abstract

**Background/Objectives:** Emerging evidence suggests that hippocampal neuroinflammation (HNF) drives cognitive decline via dysregulation of the microbiota-gut-brain axis. *Corylus heterophylla* Fisch. male flower extract (CFE), a flavonoid-rich by-product of hazelnut processing, presents a promising yet unexplored neuroprotective candidate. This study investigated the preventive effects and mechanisms of CFE against HNF-induced cognitive decline. **Methods:** In the present study, mice were pretreated with CFE (200 mg/kg) before the Lipopolysaccharide (LPS) administration. Cognitive function, inflammation, core pathology, neuroplasticity, gut microbiota and serum metabolites were assessed. The chemical composition of CFE was analyzed by UHPLC-MS and its direct immunomodulatory effects were investigated in BV2 cells. **Results:** Behavioral assessments demonstrated significant therapeutic efficacy. This was evidenced by the recovery from hippocampal damage, accompanied by reduced levels of core pathological markers (Aβ1–42, Tau, p-Tau (Ser404), GSK-3β), decreased expression of pro-inflammatory mediators including IL-33, elevated levels of neurotrophic factors (BDNF and MAP2), and attenuated abnormal activation of astrocytes and microglia. The 16S rRNA analysis confirmed that CFE ameliorated gut microbial dysbiosis. Notably, CFE significantly increased the relative abundance of *Muribaculaceae* and *Lachnospiraceae*, while significantly decreased *Staphylococcus* and *Helicobacter*. Metabolomics revealed enhanced levels of α-linolenic acid (ALA), serotonin (5-HT) and acetic acid, which correlated positively with *Muribaculaceae* and *Lachnospiraceae*. Phytochemical analysis identified luteolin and kaempferol as the predominant flavonoids in CFE. In BV2 cells, CFE, luteolin and kaempferol shifted microglial polarization from the M1 phenotype toward the M2 phenotype. **Conclusions:** CFE alleviated HNF-induced cognitive decline by regulating microbiota-gut-brain axis and microglial M1/M2 polarization.

## 1. Introduction

Cognitive decline is increasingly observed in younger populations, a trend accelerated by the COVID-19 pandemic, with hippocampal neuroinflammation (HNF) recognized as a pivotal driver in its pathogenesis [1,2]. Lipopolysaccharide (LPS)-induced HNF impairs neuronal synaptic plasticity and contributes to dysregulation across microbial, metabolic and microglial systems [3,4]. This inflammatory state facilitates the accumulation of amyloid-beta 1–42 (Aβ1–42) and promotes the aberrant phosphorylation of Tau protein [5,6]. A central kinase in this process, glycogen synthase kinase-3β (GSK-3β), drives the formation of phosphorylated Tau (P-Tau) and neurofibrillary tangles (NFTs) [7]. Concurrently, HNF triggers an upregulation of interleukin-33 (IL-33), leading to synaptic damage and a downregulation of brain-derived neurotrophic factor (BDNF) [8,9,10]. The resultant reduction in BDNF exacerbates neuronal vulnerability. Furthermore, inflammatory mediators compromise the integrity of microtubule-associated protein 2 (MAP2), disrupting neuronal structure and function, which culminates in dendritic damage and cognitive decline [11,12,13].

Peripheral inflammation can penetrate a compromised blood-brain barrier (BBB) to enter the central nervous system, thereby activating microglia [14]. Once aberrantly activated, microglia release pro-inflammatory factors (e.g., IL-1β, TNF-α, IL-33), generate reactive oxygen species (ROS), and further stimulate astrocytes, collectively amplifying HNF and driving cognitive decline [15]. The surface receptor cluster of differentiation 11b (CD11b), critical for microglial migration and phagocytosis, is significantly elevated during activation and serves as a biomarker for HNF severity [4]. As the primary immune effector cells in the brain, activated microglia polarize into pro-inflammatory (M1) and anti-inflammatory (M2) phenotypes. An imbalance in M1/M2 polarization is strongly implicated in the pathogenesis of HNF [16]. Notably, flavonoid compounds such as luteolin and kaempferol can promote a shift from the M1 to the M2 phenotype in microglial cells, characterized by the downregulation of M1 markers (iNOS, CD86, TNF-α) and upregulation of M2 markers (Arg-1, CD206, IL-10) [17,18].

The microbiota-gut-brain axis plays a significant role in modulating brain function [19]. LPS-induced gut microbial dysbiosis, marked by reduced diversity and abundance, exacerbates HNF [20]. Specifically, HNF correlates negatively with beneficial bacteria (e.g., *Muribaculaceae*, *Lachnospiraceae*) and positively with pathogenic genera (e.g., *Staphylococcus*, *Helicobacter*) [21,22,23]. Meanwhile, gut microbial metabolites, such as short-chain fatty acids (SCFAs), can cross the BBB and influence brain function, with evidence suggesting they ameliorate cognitive decline by elevating levels of BDNF and serotonin (5-HT) [24,25].

Accumulating evidence highlights the potential of floral extracts as natural neuroprotective agents against cognitive decline. For example, *Daphne genkwa* flower extract attenuated LPS-induced hippocampal neuronal loss and microglial activation by inhibiting TNF-α and IL-1β and enhancing BDNF expression [26]. *Abelmoschus Manihot Medicus* flower extract reversed cognitive decline in mice by elevating hippocampal levels of BDNF/TrkB/GluR1 [27]. *Hibiscus sabdariffa* extract improved cognitive decline by inhibiting cerebral P-Tau formation [28]. *Corylus heterophylla* Fisch., a wild hazelnut species of the family Betulaceae primarily distributed in Northeast China, notably in the Changbai Mountain region of Jilin Province, has been studied for its bioactive properties [29]. Preclinical studies indicate that the nut kernel possesses neuroprotective effects, including inhibition of Aβ generation and reduction in pro-inflammatory cytokines in the mouse hippocampus [30,31]. Pollen extracts from the male flowers of *Corylus heterophylla* Fisch. demonstrate potent antibacterial and antioxidant activities [32]. *Corylus heterophylla* Fisch. male flower extract (CFE), used traditionally for cirrhosis, demonstrates significant hepatoprotective activity against CCl4-induced liver injury in mice [33]. Furthermore, the total flavonoids from CFE protect against ischemic renal injury by mitigating oxidative stress, inhibiting profibrotic signaling, and reducing renal interstitial fibrosis [34]. However, the effects of CFE on HNF and associated cognitive decline remain unexplored. Therefore, we aimed to investigate the protective effect of CFE against cognitive decline by examining its role in regulating microbiota-gut-brain axis and microglial polarization. This research seeks to elucidate the underlying mitigating mechanisms and provide novel therapeutic strategies for cognitive decline.

## 2. Materials and Methods

### 2.1. Preparation of Flower Extract

*Corylus heterophylla* Fisch. male flowers were collected in Yanqing, Beijing. Flower samples (0.5 g) were dried in an oven, pulverized using an electric blender, and sieved (80-mesh). Ultrasonic-assisted extraction was performed using four solvent systems: (A) Methanol-water (4:6, *v*/*v*), (B) Methanol-water (8:2, *v*/*v*), (C) Chloroform-methanol (6:4, *v*/*v*), and (D) Chloroform-methanol-water (6:3:1, *v*/*v*/*v*) (10 mL each) [35,36]. Subsequently, extracts were centrifuged (10 min, 8000 rpm, 4 °C), filtered through 0.22-μm membranes, and lyophilized. The dried extracts were stored at −20 °C until further use. Three independent extractions per solvent system were conducted under identical conditions.

### 2.2. Culture and Treatment of HT22 Cells

**Figure 1:**
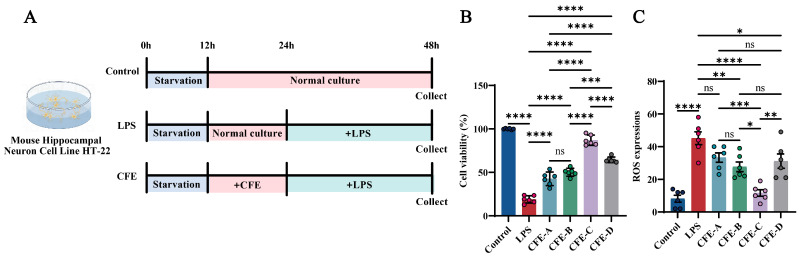
Effects of CFE on HT22 cells with lipopolysaccharide (LPS) administration. The colored dots represent data points from different sample groups. (**A**) Schematic of the experimental timeline for HT22 cell treatments. Cells were assigned to the Control, LPS and CFE groups. (**B**) Cell viability measured by CCK-8 assay. (**C**) Quantitative analysis of intracellular reactive oxygen species (ROS) levels. Data presented as means ± SEM (N = 6). Statistically significant differences were indicated: * *p* < 0.05, ** *p* < 0.01, *** *p* < 0.001, **** *p* < 0.0001, ns *p* > 0.05, compared to the LPS group.

The mouse hippocampal neuronal cell line HT22 (iCell Bioscience Inc., Shanghai, China) was cultured in Dulbecco’s Modified Eagle Medium (DMEM; cat. #11965092, Gibco, Grand Island, NY, USA) supplemented with 10% fetal bovine serum (FBS; cat. #SX1101, SORFA, Deqing, China) and 1% penicillin-streptomycin (P/S; cat. #15140122, Gibco, Grand Island, NY, USA), and maintained at 37 °C in a 5% CO_2_ atmosphere. Cells were passaged using 0.25% trypsin-0.02% EDTA (cat. #C0201, Beyotime, Shanghai, China). For viability and ROS assays, cells were seeded in 96-well plates at a density of 5 × 10^4^ cells/mL. Upon reaching 80% confluency, cells were serum-starved for 12 h and then assigned to the following groups (Figure 1A, N = 6 per group): Control: normal culture for 36 h; LPS: treated with 1 μg/mL LPS (cat. #L2880, Sigma, Kawasaki, Japan) for 24 h after 12 h of normal culture; CFE: pretreated with 200 μg/mL of solvent A/B/C/D extracts for 12 h, followed by 24 h LPS exposure. Cell viability was assessed using a CCK-8 kit (Meilunbio, Shanghai, China) by measuring the absorbance at 450 nm with an Infinite M200 microplate reader (TECAN, Männedorf, Switzerland). ROS levels were quantified with an assay kit (cat. #S0033S, Beyotime Institute of Biotechnology, Haimen, China). Briefly, cells were incubated with 10 μM 2′,7′-dichlorofluorescin diacetate (DCFH-DA) at 37 °C for 30 min. The suspensions were then centrifuged at 1000× *g* for 5 min and resuspended in fresh medium for fluorescence measurement [37].

### 2.3. The Chemical Composition of CFE Obtained with Solvent C Was Analyzed by UHPLC-MS

The chemical profile of CFE, the most effective extract obtained using solvent C, was characterized by UHPLC-MS. Chromatographic separation was carried out on a Hi Q Sil C18 column (250 mm × 4.6 mm, 5 μm) maintained at 40 °C. A gradient elution was applied over 35 min using 0.1% formic acid in water (mobile phase A) and acetonitrile (mobile phase B), at a flow rate of 0.3 mL/min. The injection volume was set to 4 μL. Mass spectrometry detection was performed in positive/negative polarity switching mode with the following parameters: sheath gas, 40 arb; auxiliary gas, 5 arb; spray voltage, 3500 V (positive) and 3000 V (negative); capillary temperature, 320 °C. Full-scan mass spectra were acquired across the m/z range of 80–1200 at a resolution of 70,000, with an AGC target of 3 × 10^6^ and a maximum injection time of 200 ms. Quality control samples were analyzed periodically to ensure system stability. Quantification of the major flavonoid compounds was conducted using the external standard method.

### 2.4. Animal Treatment

**Figure 2:**
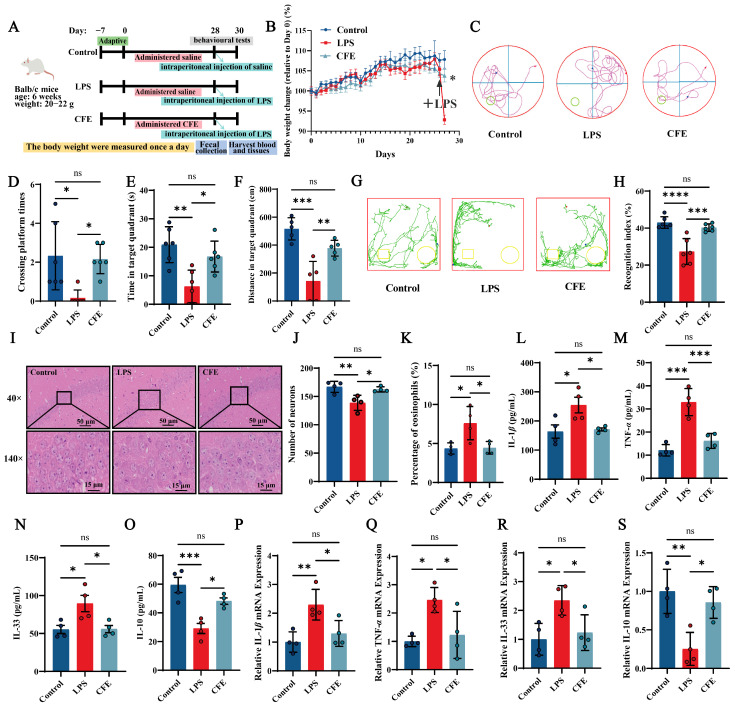
Pretreatment with *Corylus heterophylla* Fisch. male flower extract (CFE) ameliorated pathological outcomes in a mouse model of hippocampal neuroinflammation (HNF). The colored dots represent data points from different sample groups. (**A**) Schematic of the experimental timeline. (**B**) Rate of body weight change from Day 0 in mice. The arrows indicate intraperitoneal injection of LPS in mice on day 28. (**C**) Representative swimming trajectories from the Morris Water Maze test (N = 6). (**D**) Number of platform crossings within 60 s. (**E**) Time spent in the target quadrant within 60 s. (**F**) Distance traveled in the target quadrant within 60 s. (**G**) Representative exploration curves from the novel object recognition test (N = 6). (**H**) Recognition index for each group. (**I**) Representative hematoxylin and eosin (H&E)-stained sections of the hippocampus. (**J**) Quantification of neuronal density in the hippocampal cornu ammonis 1 (CA1) region. (**K**) Percentage of eosinophilic cells in the CA1 region. (**L**–**O**) Serum levels of IL-1β, TNF-α, IL-33, and IL-10 measured by ELISA. (**P**–**S**) Hippocampal mRNA expression of IL-1β, TNF-α, IL-33, and IL-10 assessed by RT-qPCR. Data are presented as mean ± SEM (N = 4). Statistically significant differences were indicated: * *p* < 0.05, ** *p* < 0.01, *** *p* < 0.001, **** *p* < 0.0001, ns *p* > 0.05, compared to the LPS group.

All animal procedures were approved by the Animal Experimentation Ethics Committee of China Agricultural University (Protocol Aw71705202-6-04, 17 July 2025). As outlined in Figure 2A, a total of 30 male Balb/c mice (6 weeks old, 20–22 g; Beijing Sibeifu Biotechnology Co., Ltd., Beijing, China) were randomly assigned to three groups: Control, LPS and CFE. Following a 7-day acclimatization period, mice received daily intragastric gavage for 28 consecutive days. The Control and LPS groups were administered 0.2 mL of saline per day, whereas the CFE group received 0.2 mL of CFE (extracted with solvent C, 200 mg/kg) daily. The CFE dose was selected based on preliminary experiments and previous literature [38]. Upon completion of the 28-day gavage regimen, all mice except the Control group received a single intraperitoneal injection of LPS (1 mg/kg). Control mice were injected with an equal volume of saline. Body weight was recorded daily throughout the study.

### 2.5. Behavioral Assessments

#### 2.5.1. Morris Water Maze (MWM)

Spatial learning and memory were assessed using the MWM test [37]. The apparatus consisted of a circular pool (120 cm in diameter) filled with water maintained at 25 °C. A submerged escape platform (10 cm in diameter, positioned 2 cm below the water surface) was placed in the center of quadrant III. During the training phase, mice that failed to locate the platform within 60 s were gently guided to it and allowed to remain there for 15 s. For the probe test, the platform was removed and each mouse was released into quadrant I. The following parameters were recorded over a 60 s period: the number of platform crossings, and the time spent and distance traveled in the target quadrant.

#### 2.5.2. Novel Object Recognition Experiment (NOR)

In the NOR test, during the training phase, two identical rectangular objects (A and B) were placed in opposite corners of the arena. Mice were positioned facing away from the objects to avoid initial bias and allowed to explore freely for 5 min. In the subsequent test phase, object B was replaced with a novel cylindrical object (C). Mice were reintroduced into the arena, and their exploration was recorded for 5 min. The following parameters were quantified: movement trajectories, exploration time directed toward the familiar object A (T_A_), and exploration time directed toward the novel object C (T_C_) [39]. The recognition index was calculated as follows: Recognition Index (%) = [T_C_/(T_A_ + T_C_)] × 100%.

### 2.6. Hematoxylin and Eosin (H&E) Staining

Following behavioral testing, mice were transcardially perfused with saline followed by 4% paraformaldehyde. Brains were promptly dissected and further fixed by immersion in 4% PFA for 24 h at 4 °C. After graded ethanol dehydration, the tissues were paraffin embedded using a tissue embedding station. Subsequently, 4-μm-thick sections were cut and prepared for H&E staining. To evaluate neuronal damage in the hippocampal cornu ammonis 1 (CA1) region, we quantified the density of surviving neurons and the proportion of eosinophilic cells [40]. Analysis was conducted on four randomly selected 40× fields per section using ImageJ2x software (Rawak Software, Stuttgart, Germany).

### 2.7. Enzyme-Linked Immunosorbent Assay (ELISA)

Blood samples were collected from all mice (Control, LPS and CFE groups) via retro-orbital bleeding upon completion of behavioral testing, which was performed 2 days after the final LPS challenge and pretreatment administration. Samples were centrifuged at 3000× *g* for 15 min at 4 °C, and the serum was collected for analysis. Serum concentrations of IL-1β (Cat. #RX203063M, RUIXIN, Yueqing, China), TNF-α (Cat. #EK282, RUIXIN), IL-33 (Cat. #RX203053M, RUIXIN), IL-10 (Cat. #EK210, RUIXIN), 5-HT (Cat. #MM-0443M2, Jiangsu Meimian Industrial Co., Ltd., Yancheng, China), and ALA (Cat. #MM-926718O2, Jiangsu Meimian Industrial Co., Ltd.) were quantified using commercially available ELISA kits [41]. For hippocampal tissue analysis, 20 mg of tissue was homogenized in 1 mL of RIPA buffer (MA0151, Meilunbio) supplemented with protease inhibitors. The homogenate was centrifuged at 10,000× *g* for 5 min at 4 °C, and the resulting supernatant was collected. Hippocampal levels of 5-HT and ALA were measured using specific ELISA kits, with absorbance read on a microplate reader (model 3903 2010, Bio Rad, Hercules, CA, USA).

### 2.8. Quantitative Real-Time Polymerase Chain Reaction (qRT-PCR)

Total RNA was isolated from hippocampal tissue using TRIzol™ Reagent (Cat. #15596026, Invitrogen, Carlsbad, CA, USA). RNA quality and concentration were assessed using a NanoDrop 2000 spectrophotometer (Thermo Fisher Scientific, Shanghai, China). Complementary DNA (cDNA) was synthesized using the PrimeScript RT Reagent Kit (Cat. #DRR037A, TaKaRa, Osaka, Japan). qRT-PCR was then performed with TB Green Premix Ex Taq (Cat. #RR420A, Takara) on a real-time PCR system. Gene expression levels were calculated using the 2^−ΔΔCt^ method and normalized to the housekeeping gene GAPDH. The primer sequences used are listed in Supplementary Table S1.

### 2.9. Immunohistochemistry (IHC)

IHC staining of brain tissue sections was performed using primary antibodies against the following targets: Aβ1–42 (cat. #A9810, Sigma Aldrich, St. Louis, MO, USA), Tau (cat. #10274-1-AP, Proteintech, Rosemont, IL, USA), P-Tau (Ser404) (cat. #35834, Cell Signaling Technology, Danvers, MA, USA), BDNF (cat. #25699-1-AP, Proteintech), MAP2 (cat. #17490-1-AP, Proteintech) and CD11b (cat. #66519-1-IG, Proteintech). Antigen antibody complexes were detected using a horseradish peroxidase-conjugated secondary antibody followed by diaminobenzidine chromogenic development with a Vectastain ABC kit [42]. The average optical density (AOD) of immunopositive regions was quantified in four randomly selected fields within the hippocampal CA1 subregion using ImageJ2x software (Rawak Software, Germany).

### 2.10. Western Blot Analysis (WB)

Western blot analysis was performed following a previously described protocol with slight modifications [43]. Hippocampal tissues were homogenized on ice for 30 min in RIPA lysis buffer supplemented with 1% PMSF and phosphatase inhibitors (Beyotime, Shanghai, China). After centrifugation at 12,000× *g* for 20 min at 4 °C, the supernatant was collected, and total protein concentration was determined using a bicinchoninic acid (BCA) assay kit (Beyotime, China). Protein samples were denatured by heating at 95 °C for 20 min, separated on 10% SDS-polyacrylamide gels, and transferred onto polyvinylidene fluoride (PVDF) membranes (cat. #IPVH00010, Millipore, Burlington, MA, USA). Membranes were blocked with 5% bovine serum albumin (BSA) in PBS for 1 h at room temperature and then incubated overnight at 4 °C with the following primary antibodies: Aβ1–42 (cat. #A9810, Sigma-Aldrich), Tau (cat. #10274-1-AP, Proteintech), P-Tau (Ser404) (cat. #35834, Cell Signaling Technology, Danvers, MA, USA), GSK-3β (cat. #22104-1-AP, Proteintech), BDNF (cat. #25699-1-AP, Proteintech), MAP2 (cat. #17490-1-AP, Proteintech), CD11b (cat. #ab184307, Abcam, Cambridge, UK), and GAPDH (cat. #10494-1-AP, Proteintech). After washing, membranes were incubated for 2 h at room temperature with an HRP-conjugated secondary antibody (cat. #SA00001 2, Proteintech). Protein bands were visualized using enhanced chemiluminescence (ECL) substrate on an Image Quant LAS4000 system (GE Healthcare, Chicago, IL, USA). Band intensities were quantified using ImageJ2x software (National Institutes of Health, Bethesda, MD, USA) and normalized to GAPDH.

### 2.11. Immunofluorescence (IF)

Brain tissues were fixed in 4% paraformaldehyde, embedded, and sectioned to a thickness of 40 μm using a cryostat. After three brief washes with PBS, sections were blocked with 5% BSA for 1 h at room temperature and then incubated overnight at 4 °C with the following primary antibodies: rabbit anti-Iba1 (1:500, ab178846, Abcam) and rabbit anti-GFAP (1:500, ab7260, Abcam). Following three PBS washes, sections were incubated with appropriate fluorescent dye conjugated secondary antibodies for 2 h at room temperature in the dark. After a final PBS wash, sections were mounted, air dried, and imaged using a laser scanning confocal microscope (Thermo Fisher Scientific, Waltham, MA, USA).

### 2.12. Metabolomic Profiling of Fecal Short-Chain Fatty Acids (SCFAs)

Quantitative analysis of fecal SCFAs (acetic, propionic and butyric acids) was performed by targeted metabolomics following an established methodology [44]. Fecal samples (25 mg) were subjected to acid extraction using 0.5% phosphoric acid (H_3_PO_4_), followed by cryogenic homogenization, sonication in an ice bath for 10 min, and centrifugation. The supernatant was derivatized with butanol containing 2-ethylbutyric acid as an internal standard, then vortexed, sonicated, and centrifuged. GC-MS analysis was carried out on an Agilent 8890B/5977B system equipped with an HP-FFAP column (30 m × 0.25 mm × 0.25 μm). The operating conditions were as follows: splitless injection at 260 °C; helium carrier gas at a constant flow of 1.0 mL/min; oven temperature programmed from 80 °C (held for 1 min) to 120 °C at 40 °C/min, then to 200 °C at 10 °C/min, and finally to 230 °C (held for 3 min). Detection was performed in selected ion monitoring (SIM) mode using electron ionization (70 eV). The ion source and transfer line temperatures were set to 230 °C, and the quadrupole temperature was 150 °C. Quantification was based on external calibration curves generated with MassHunter software (v10.0).

### 2.13. Sequencing of the Microbial 16S Ribosomal RNA (16S rRNA)

Fecal samples collected from the experimental mice were immediately frozen at −80 °C and processed within 3 h. DNA extraction and 16S rRNA gene sequencing were subsequently conducted by Majorbio Bio Pharm Technology Co., Ltd. (Shanghai, China). Briefly, the V3–V4 hypervariable regions of the bacterial 16S rRNA gene were amplified and sequenced on an Illumina NextSeq 2000 platform. After DNA extraction and PCR amplification, the raw sequencing reads were subjected to quality filtering, paired end assembly, and clustering into operational taxonomic units (OTUs). Microbial diversity was analyzed with beta diversity assessed via principal coordinate analysis (PCoA) based on Bray-Curtis distances. Differentially abundant bacterial genera were identified using linear discriminant analysis effect size (LEfSe) with thresholds of LDA score > 3 and *p* < 0.05.

### 2.14. Serum Metabolomics Analysis

Untargeted metabolomic profiling of serum samples was conducted following an adapted protocol [45]. Briefly, serum proteins were precipitated using cold methanol acetonitrile (1:1, *v*/*v*). After centrifugation, the supernatant was collected, dried under nitrogen, reconstituted in an appropriate solvent, and analyzed on a UHPLC-MS/MS system (Thermo Scientific Q Exactive, Waltham, MA, USA). Chromatographic separation was performed on an HSS T3 column using a gradient of water and acetonitrile, each containing 0.1% formic acid. Mass spectrometry analysis was carried out in data dependent acquisition (DDA) mode with electrospray ionization in both positive and negative polarities at a resolution of 70,000.

### 2.15. Culture and Treatment of BV2 Cells

BV2 microglial cells were maintained in complete DMEM and seeded in 6 well plates at a density of 5 × 10^4^ cells/mL. Upon reaching 80% confluency, cells were serum starved for 12 h. In a preliminary experiment, the optimal CFE concentration was determined using the following groups: Control (normal culture), LPS (1 μg/mL, 12 h normal culture + 24 h LPS) and CFE (200, 400, or 600 μg/mL; 12 h pretreatment with CFE + 24 h LPS). Based on cell viability results, 400 μg/mL CFE was selected for subsequent polarization studies. For M1 polarization, the experimental groups included: Control (normal culture), LPS (1 μg/mL, 12 h normal culture + 24 h LPS), CFE (400 μg/mL, 12 h pretreatment with CFE + 24 h LPS), luteolin (14 μM, CAS: 491-70-3, cat. #B20888, Shanghai Yuanye BioTechnology Co., Ltd., Shanghai, China, 12 h pretreatment with luteolin + 24 h LPS) and kaempferol (10 μM, CAS: 520-18-3, cat. #YZ-110861, Solarbio, Beijing, China, 12 h pretreatment with kaempferol + 24 h LPS). For M2 polarization, groups included: Control (normal culture), IL-4 (20 ng/mL, cat. #P00196, Solarbio, 12 h normal culture + 24 h IL-4), CFE (400 μg/mL, 12 h pretreatment with CFE+ 24 h IL-4), luteolin (14 μM, 12 h pretreatment with luteolin + 24 h IL-4) and kaempferol (10 μM, 12 h pretreatment with kaempferol + 24 h IL-4). Total RNA was extracted using TRIzol reagent, and cDNA was synthesized. mRNA expression levels of M1 markers (iNOS, CD86, TNF-α) and M2 markers (Arg1, CD206, IL-10) were analyzed by quantitative PCR using the 2^−ΔΔCt^ method (primer sequences are listed in Supplementary Table S1).

### 2.16. Data Processing

Data are presented as mean ± standard error of the mean (SEM). Statistical analyses were performed with Prism 9.5 (GraphPad Software, San Diego, CA, USA). Specifically, one-way analysis of variance (ANOVA) was applied, followed by Tukey’s post hoc test for multiple comparisons. Differences were considered statistically significant at *p* < 0.05.

## 3. Results

### 3.1. CFE Enhanced Cell Viability and Attenuates ROS Production in HT22 Cells

We evaluated the effects of CFE on cell viability and ROS levels in HT22 cells using an LPS-induced model of HNF (Figure 1A–C). The CCK-8 assay revealed that LPS treatment significantly decreased HT22 cell viability compared to the Control group (*p* < 0.0001). This decrease was significantly mitigated by pretreatment with CFE-A, CFE-B, CFE-C and CFE-D (200 μg/mL, all *p* < 0.0001). Furthermore, CFE exhibited antioxidant activity, as LPS-induced ROS overproduction (*p* < 0.0001) was significantly suppressed by CFE-B (*p* < 0.001), CFE-C (*p* < 0.0001) and CFE-D (*p* < 0.05), with the solvent C extract showing the most pronounced reduction. Based on the combined neuroprotective and antioxidant effects, CFE extracted with solvent C was selected for all subsequent experiments.

### 3.2. Metabolite Profiling of CFE Extracted with Solvent C

Metabolite analysis of the solvent C extract of CFE tentatively identified 274 compounds, which were categorized into the following chemical classes: 91 terpenoids, 75 flavonoids, 52 phenylpropanoids, 20 alkaloids, 9 polyketides, 9 shikimate/acetate-malonate pathway derived compounds, 7 fatty acid-related compounds, 7 amino acid-related compounds, and 4 others. The ten most abundant and biologically relevant compounds from each major class are listed in Table 1, along with their tentative identities, molecular formulas, retention times, and *m*/*z* values. Subsequently, targeted quantitative analysis of the ten principal flavonoids was performed by HPLC-MS/MS (Table 2). Among these, luteolin and kaempferol were the most abundant, with quantified concentrations of 10,796.37 ± 65.53 ng/mL and 3082.77 ± 110.12 ng/mL, respectively.

### 3.3. CFE Attenuated Cognitive Decline in HNF Mice

To evaluate the potential of CFE in mitigating LPS-induced HNF and cognitive decline, mice were administered CFE for four weeks (Figure 2A). No significant differences in body weight were observed among groups prior to LPS challenge. Following LPS administration, mice in the LPS group exhibited significant weight loss, confirming successful model induction. Notably, CFE pretreatment significantly attenuated this LPS-induced weight reduction (*p* < 0.05), indicating its ability to counteract inflammation-associated metabolic changes (Figure 2B).

Spatial learning and memory were assessed using the MWM (Figure 2C–F). Compared with the Control group, LPS-treated mice showed significant decreases in the number of platform crossings (*p* < 0.05), time spent in the target quadrant (*p* < 0.01), and distance traveled in the target quadrant (*p* < 0.001), indicating marked spatial memory deficits. CFE pretreatment significantly improved these parameters, increasing platform crossings (*p* < 0.05), time in the target quadrant (*p* < 0.05), and distance traveled in the target quadrant (*p* < 0.01) relative to the LPS group, demonstrating that CFE alleviates spatial memory impairment.

These findings were further supported by the NOR test (Figure 2G,H). Mice pretreated with CFE displayed a significantly higher recognition index than LPS-treated mice (*p* < 0.001), reflecting enhanced exploratory behavior and novel-object discrimination. Collectively, these results indicate that CFE pretreatment effectively ameliorates the severe cognitive decline associated with HNF in mice.

### 3.4. CFE Alleviated Hippocampal Damage in HNF Mice

Hippocampal histopathological changes are presented in Figure 2I–K. The LPS group showed a disrupted cytoarchitecture characterized by disorganized neuronal arrangement, widened intercellular spaces, and irregular morphology. Nucleolar condensation, dissolution, or loss was also evident. Quantitative analysis revealed a significant reduction in neuronal density (*p* < 0.01) and in the proportion of eosinophilic neurons (*p* < 0.05) compared to the Control group. In contrast, CFE intervention effectively restored hippocampal histology, significantly increasing both neuronal density (*p* < 0.05) and the percentage of eosinophilic neurons (*p* < 0.05) relative to the LPS group. Together, these results demonstrate that CFE pretreatment confers substantial protection against HNF-induced structural damage in the hippocampus.

### 3.5. CFE Modulated Inflammatory Cytokine Levels in Serum and Hippocampus of HNF Mice

ELISA analysis of serum (Figure 2L–O) showed that LPS administration significantly increased the levels of the pro-inflammatory cytokines IL-1β (*p* < 0.05), TNF-α (*p* < 0.001), and IL-33 (*p* < 0.05), while reducing the anti-inflammatory cytokine IL-10 (*p* < 0.001). In contrast, CFE pretreatment significantly lowered serum IL-1β (*p* < 0.05), TNF-α (*p* < 0.001), and IL-33 (*p* < 0.05), and elevated IL-10 (*p* < 0.05) compared to the LPS group. Similarly, RT-qPCR analysis of hippocampal tissue (Figure 2P–S) indicated that LPS challenge markedly upregulated mRNA expression of IL-1β (*p* < 0.01), TNF-α (*p* < 0.05), and IL-33 (*p* < 0.05), and downregulated IL-10 (*p* < 0.01). CFE intervention significantly reversed these changes, reducing IL-1β, TNF-α, and IL-33 expression and enhancing IL-10 levels (all *p* < 0.05). Together, these data demonstrate that CFE effectively mitigates systemic and hippocampal inflammation in HNF mice.

### 3.6. CFE Alleviated the Expression of Aβ1–42 Protein in the Hippocampus of HNF Mice

**Figure 3:**
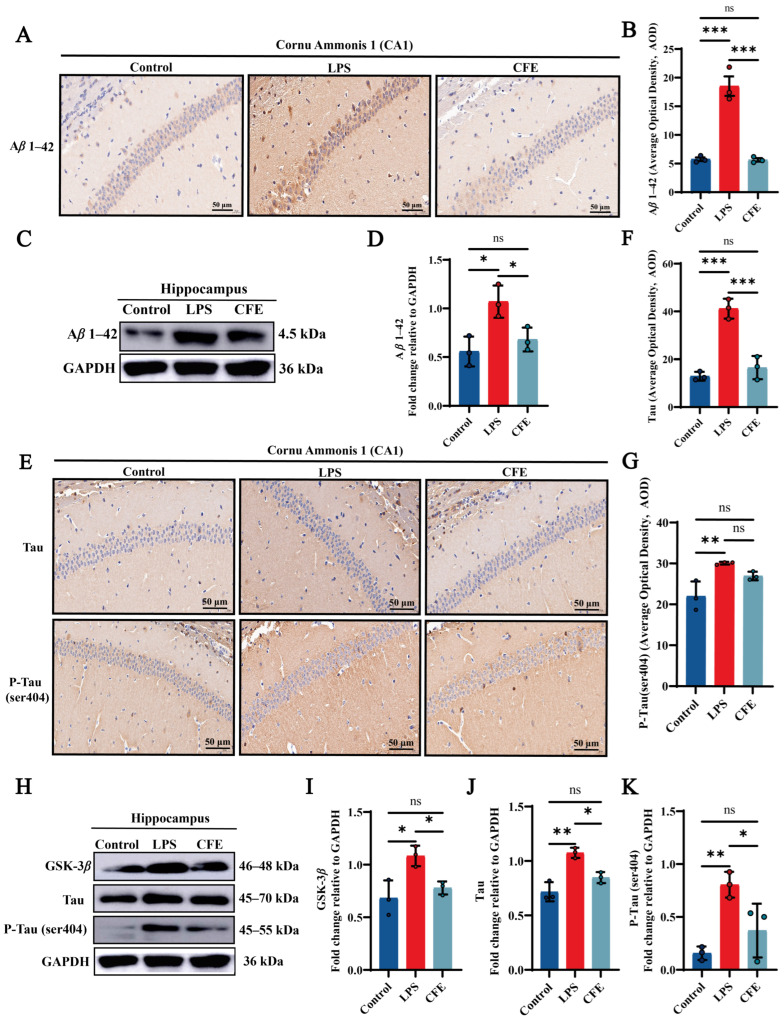
CFE downregulated hippocampal levels of Aβ1–42, GSK-3β, Tau and P-Tau in HNF mice. The colored dots represent data points from different sample groups. (**A**) Representative immunohistochemical (IHC) staining images of Aβ1–42 (40× magnification). (**B**) Quantification of Aβ1–42 IHC staining intensity expressed as average optical density (AOD). (**C**) Representative Western blot images of hippocampal Aβ1–42. (**D**) Quantitative analysis of Aβ1–42 protein levels from Western blot, normalized to GAPDH. (**E**) Representative IHC staining images of total Tau and P-Tau (Ser404) (40×). (**F**,**G**) Quantification of Tau and P-Tau (Ser404) IHC staining intensity (AOD). (**H**) Representative Western blot images of GSK-3β, total Tau and P-Tau (Ser404). (**I**–**K**) Quantitative analysis of GSK-3β, total Tau and P-Tau (Ser404) protein levels from Western blot, normalized to GAPDH. Data are presented as the mean ± SEM (N = 3). Statistically significant differences were indicated: * *p* < 0.05, ** *p* < 0.01, *** *p* < 0.001, ns *p* > 0.05, compared to the LPS group.

To assess the impact of CFE on Aβ1–42 levels, IHC (Figure 3A,B) and WB (Figure 3C,D) were performed. IHC analysis confirmed that the LPS group exhibited significantly elevated AOD values for Aβ1–42 compared to the Control group (*p* < 0.001), as evidenced by widespread brown-yellow deposition throughout the hippocampus. CFE intervention markedly reduced Aβ1–42 AOD relative to the LPS group (*p* < 0.001). WB analysis confirmed this reduction (*p* < 0.05). The results suggested that the amelioration of cognitive decline by CFE in HNF mice is associated with reduced hippocampal Aβ1–42 deposition and subsequent improvement in the brain microenvironment.

### 3.7. CFE Alleviated the Expressions of GSK-3β, Tau and P-Tau Protein in the Hippocampus of HNF Mice

IHC and WB analysis of CA1 hippocampal regions quantified GSK-3β, Tau and P-Tau (ser404) immunoreactivity (Figure 3E–K). Compared to the Control group, the LPS group demonstrated a marked increase in AOD values for Tau (*p* < 0.001) and P-Tau (ser404) (*p* < 0.01), characterized by extensive brown-yellow deposits diffusely distributed across the hippocampal formation. CFE intervention significantly lowered Tau (*p* < 0.001) and P-Tau (ser404) AOD value compared to the LPS group. Furthermore, WB analysis revealed a decrease in GSK-3β, Tau and P-Tau (ser404) (all *p* < 0.05) expression which is consistent with the IHC results. Our results show that CFE reduces hippocampal GSK-3β, Tau and P-Tau (ser404) expression, suggesting a potential mechanism for neuronal cytoskeletal stabilization.

### 3.8. CFE Enhanced the Expressions of BDNF and MAP2 Protein in the Hippocampus of HNF Mice

**Figure 4:**
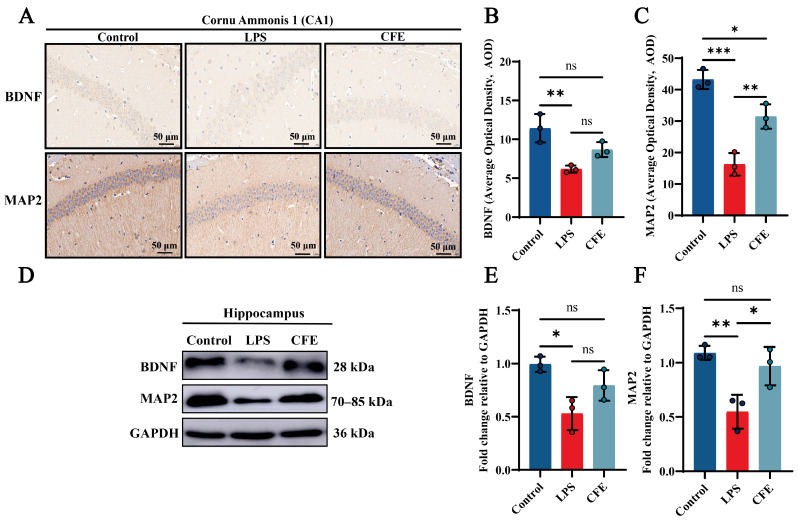
CFE increased hippocampal levels of BDNF and MAP2 in HNF mice. The colored dots represent data points from different sample groups. (**A**) Representative immunohistochemical (IHC) staining images of BDNF and MAP2. (**B**,**C**) Quantification of BDNF and MAP2 IHC staining intensity expressed as AOD. (**D**) Representative Western blot images of hippocampal BDNF and MAP2. (**E**,**F**) Quantitative analysis of BDNF and MAP2 protein levels from Western blot, normalized to GAPDH. Data are presented as the mean ± SEM (N = 3). Statistically significant differences were indicated: * *p* < 0.05, ** *p* < 0.01, *** *p* < 0.001, ns *p* > 0.05, compared to the LPS group.

IHC examination of CA1 hippocampal subfields was performed to quantify BDNF and MAP2 expression (Figure 4A–C). In the Control group, BDNF and MAP2 high expressions were a brown-yellow sediment, which was widely distributed in the hippocampus. CFE supplementation significantly increased BDNF and MAP2 AOD value compared to the LPS group (*p* < 0.01). Notably, WB analysis (Figure 4D–F) revealed enhanced expression of both BDNF and MAP2 in the CFE group compared to the LPS group (*p* < 0.05). Our findings indicated that CFE enhanced neuronal synaptic plasticity by modulating the BDNF signaling pathway.

### 3.9. CFE Inhibited the Abnormal Activation of Microglia and Astrocytes in the Hippocampus of HNF Mice

**Figure 5:**
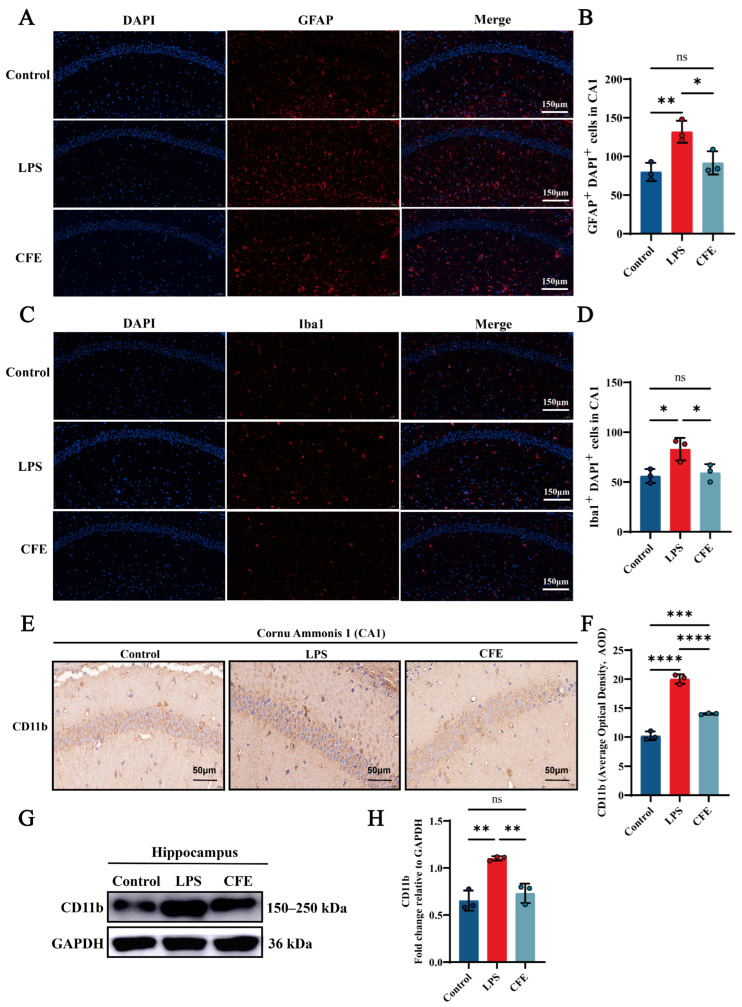
CFE suppressed glial cell activation in the hippocampus of HNF mice. The colored dots represent data points from different sample groups. (**A**) Representative immunofluorescence (IF) image of GFAP (astrocyte marker) in the hippocampal CA1 region. (**B**) Quantitative analysis of GFAP immunofluorescence intensity. (**C**) Representative immunofluorescence image of Iba1 (microglial marker) in the hippocampal CA1 region. (**D**) Quantitative analysis of Iba1 immunofluorescence intensity. (**E**) Representative immunohistochemical (IHC) staining of CD11b. (**F**) Quantification of CD11b IHC intensity expressed as AOD. (**G**) Representative Western blot images of hippocampal CD11b. (**H**) Quantitative analysis of CD11b protein levels from Western blot, normalized to GAPDH. Data are presented as the mean ± SEM (N = 3). Statistically significant differences were indicated: * *p* < 0.05, ** *p* < 0.01, *** *p* < 0.001, **** *p* < 0.0001, ns *p* > 0.05, compared to the LPS group.

IF analysis revealed that the activation of microglia and astrocytes, as indicated by Iba1 and GFAP labeling, respectively, was significantly attenuated in the CFE group compared to the LPS group (all *p* < 0.05) (Figure 5A–D). Additionally, IHC analysis demonstrated that CFE intervention markedly reduced CD11b AOD compared to the LPS group (*p* < 0.0001) (Figure 5E,F). Concordantly, WB analysis (Figure 5G,H) confirmed decreased CD11b protein expression in the CFE group (*p* < 0.01). These data collectively indicated that CFE inhibited microglial and astrocyte activation.

### 3.10. CFE Increased SCFAs in Fecal of HNF Mice

**Figure 6:**
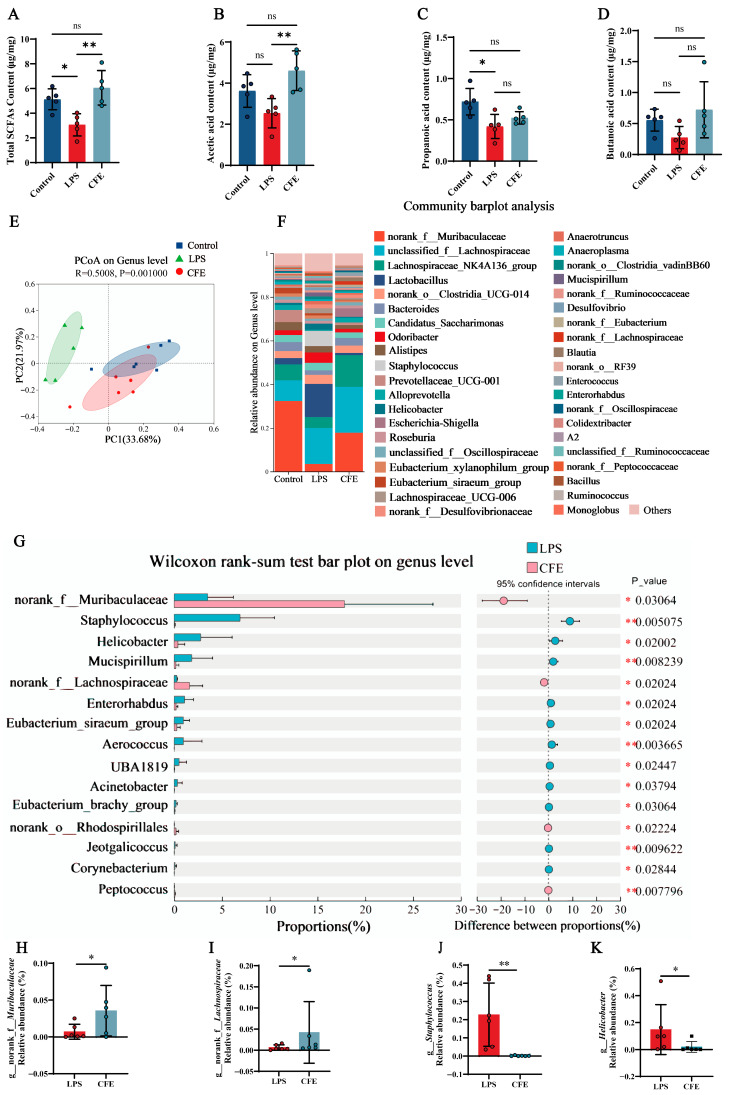
CFE modulates the gut microbiome and enhances SCFAs production in HNF mice. The colored dots represent data points from different sample groups. (**A**) Fecal concentration of total SCFAs (N = 5). (**B**) Fecal concentration of acetic acid (N = 5). (**C**) Fecal concentration of propionic acid (N = 5). (**D**) Fecal concentration of butyric acid (N = 5). (**E**) Beta diversity of the gut microbiota visualized by PCoA based on Bray-Curtis distances at the genus level. The plot shows the microbial community composition of samples from the Control (blue), LPS (green), and CFE (red) groups. Each dot represents an individual sample. The background color illustrates the clustering of samples within the ordination space. (**F**) Bar plot showing the relative abundance of bacterial genera. (**G**) Differential abundance analysis between the CFE and LPS groups (Wilcoxon rank-sum test). (**H**) Relative abundance of the genus *Muribaculaceae* across groups. (**I**) Relative abundance of the genus *Lachnospiraceae* across groups. (**J**) Relative abundance of the genus *Staphylococcus* across groups. (**K**) Relative abundance of the genus *Helicobacter* across groups. Data are presented as the mean ± SEM (N = 6). Statistically significant differences were indicated: * *p* < 0.05, ** *p* < 0.01, ns *p* > 0.05, compared to the LPS group.

Given the established role of SCFAs in microbiota-gut-brain axis signaling, we measured fecal SCFAs levels to evaluate the effect of CFE on gut microbiota metabolism (Figure 6A–D). Notably, CFE intervention elevated total SCFAs (*p* < 0.01), acetic acid (*p* < 0.01), propanoic acid and butanoic acid compared to the LPS group. The results indicated that CFE reversed the reduction in fecal SCFAs levels induced by HNF.

### 3.11. CFE Reshaped the Gut Microbiota of HNF Mice

Fecal 16S rRNA gene sequencing was performed to evaluate CFE’s impact on gut microbiota in HNF mice. PCoA based on Bray-Curtis distances (Figure 6E) revealed distinct inter-group clustering. PC1 and PC2 accounted for 33.68% and 21.97% of variance, respectively. Notably, reduced compositional overlap between the LPS and CFE groups indicated microbiota restructuring. At the genus level (Figure 6F), CFE administration shifted dominant bacterial flora from *Lactobacillus* and *Staphylococcus* (LPS group) to *norank_f__Muribaculaceae* and *unclassified_f__Lachnospiraceae* (CFE group). Wilcoxon tests (Figure 6G–K) confirmed CFE significantly increased *norank_f__Muribaculaceae* (*p* < 0.05) and *norank_f__Lachnospiraceae* (*p* < 0.05), while decreasing *Staphylococcus* (*p* < 0.01) and *Helicobacter* (*p* < 0.05) compared to the LPS group. Collectively, the findings proved that CFE supplementation renovated LPS-induced gut microbiota disturbances.

### 3.12. CFE Regulated Serum Metabolites in HNF Mice

**Figure 7:**
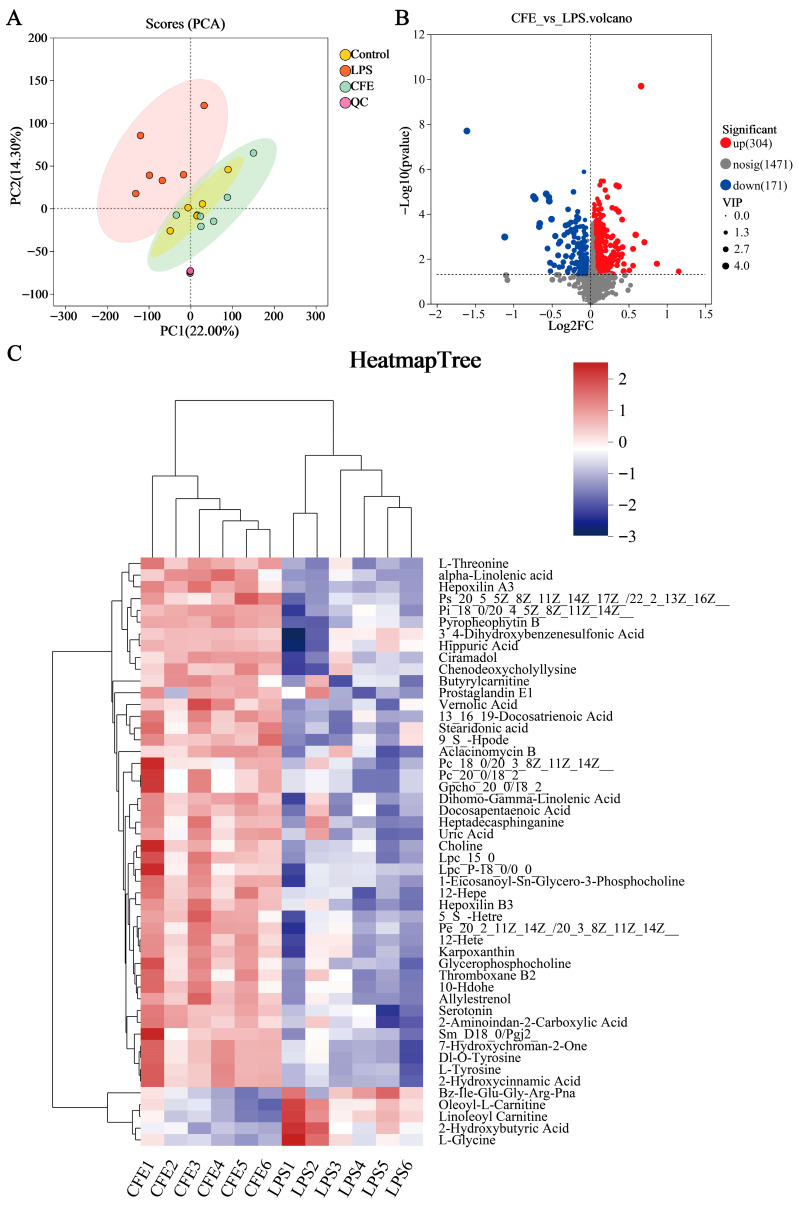

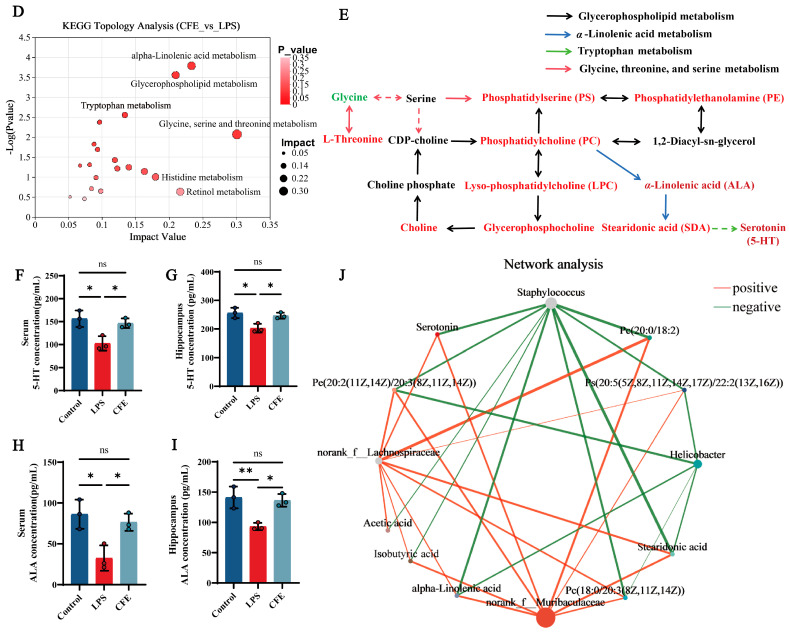
CFE modulated host systemic metabolism. The colored dots represent data points from different sample groups. (**A**) Principal component analysis (PCA) score chart. (**B**) The volcano plots of significantly differential metabolic components in the CFE group compared to the LPS group (*p* < 0.05). (**C**) Hierarchical cluster analysis of differential metabolites between the CFE group and the LPS group. (**D**) Potential target metabolic pathways of CFE in the pretreatment of HNF mice. (**E**) A schematic map depicting the enriched key pathways and the targeted compounds regulated by CFE in HNF mice; Red represents the upregulation of metabolites and green represents the downregulation of metabolites. (**F**,**G**) 5-HT concentrations in serum and hippocampus (N = 3). (**H**,**I**) ALA concentrations in serum and hippocampus (N = 3). (**J**) Integrated analysis reveals that the gut microbiome is associated with changes in metabolites. Red edges indicate a positive correlation and green edges indicate a negative correlation. Data are presented as the means ± SEM (N = 6). Statistically significant differences were indicated: * *p* < 0.05, ** *p* < 0.01, ns *p* > 0.05, compared to the LPS group.

Serum untargeted metabolomic profiling was employed to characterize systemic metabolic alterations during HNF progression. The clear separation between the LPS and control groups in the PCA score plot (Figure 7A) confirmed distinct metabolic profiles and verified successful model induction. Differential metabolites were identified using dual thresholds of variable importance in projection (VIP) > 1 and statistical significance (*p* < 0.05), with fold-change (FC) values determining regulation directionality (FC < 1: downregulation; FC > 1: upregulation; Figure 7B). 475 metabolites exhibited significant abundance changes between the LPS and CFE groups (304 upregulated, 171 downregulated). Hierarchical clustering analysis revealed that the CFE group substantially increased concentrations of phosphatidylserine [PS (20:5 (5Z,8Z,11Z,14Z,17Z)/22:1 (13Z))], phosphatidylcholines [PC (18:0/20:3 (8Z,11Z,14Z), PC (20:0/18:2)], phosphatidylethanolamine [PE (20:2 (11Z,14Z)/20:3 (8Z,11Z,14Z))], lysophosphatidylcholines [LPC (15:0), LPC (P-18:0/0:0)], choline, ALA, stearidonic acid (SDA), and 5-HT compared to the LPS group (Figure 7C). KEGG Topological analysis further identified 20 significantly enriched metabolic routes, notably encompassing glycerophospholipid metabolism, α-linolenic acid metabolism, tryptophan metabolism, and glycine-serine-threonine metabolism (Figure 7D). Metabolomic interaction networks revealed the pathway interconnections and underlying mechanisms (Figure 7E). ELISA quantification confirmed CFE group elevations of 5-HT (*p* < 0.05) and ALA (*p* < 0.05) in both serum and hippocampal tissues compared to the LPS group (Figure 7F–I). For establishing gut-microbiota-metabolite crosstalk, Pearson correlation-based networks were generated and genus-level networks demonstrated significant associations (Figure 7J): g__norank_f__*Muribaculaceae* and g__norank_f__*Lachnospiraceae* abundances showed positive correlations with PC (15:0/18:2), PC (20:0/18:2), PE (20:2/20:3), PS (20:5/22:1), 5-HT, ALA, SDA, acetate, and isobutyrate, while *Staphylococcus* abundance was inversely correlated with these metabolites. Similarly, *Helicobacter* exhibited negative correlations specifically with PC (18:0/20:3), PE (20:2/20:3), PS (20:5/22:1), ALA, and SDA. Collectively, CFE mediated microbiota remodeling characterized by enrichment of *Muribaculaceae* and *Lachnospiraceae* alongside suppression of *Staphylococcus* and *Helicobacter* potentiated beneficial metabolite production. 

### 3.13. CFE, Luteolin, and Kaempferol Significantly Improved Cell Viability and Reduced ROS Levels in BV2 Cells

**Figure 8:**
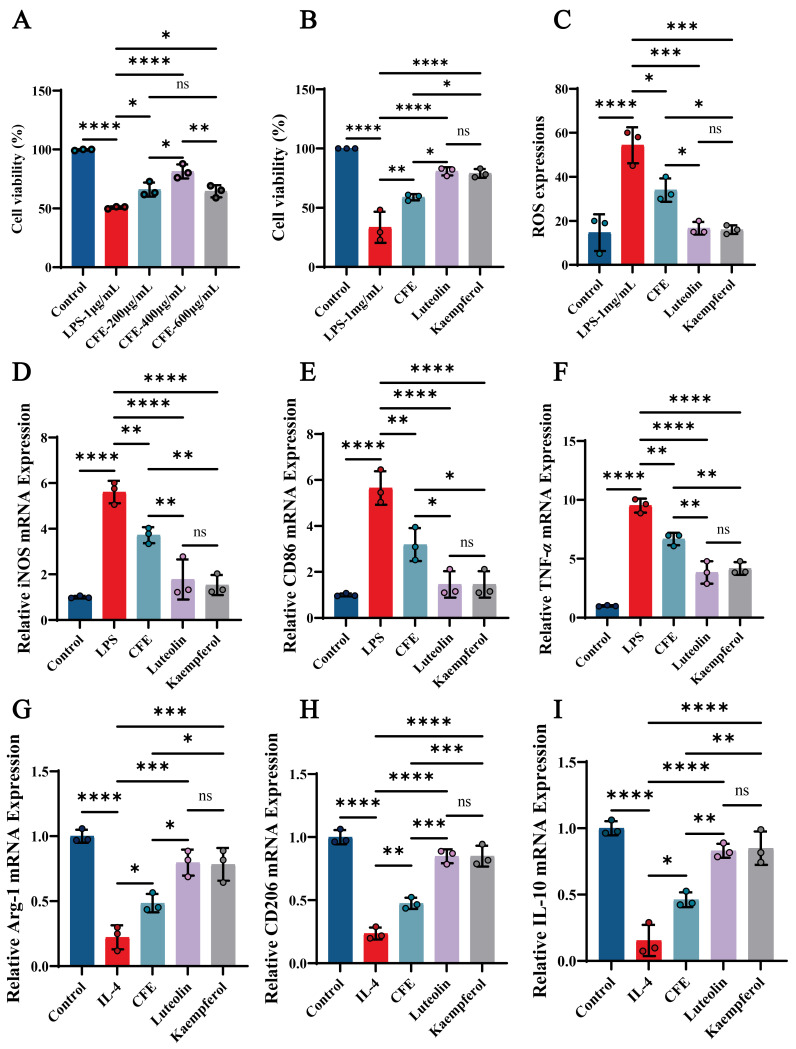
CFE regulated microglial polarization in LPS-induced BV2 cells. The colored dots represent data points from different sample groups. (**A**) Cell viability assessed by CCK-8 assay following pretreatment with increasing concentrations of CFE. (**B**) Cell viability measured by CCK-8 after pretreatment with CFE, luteolin or kaempferol. (**C**) Quantitative analysis of intracellular ROS levels. (**D**–**F**) mRNA expression of M1 polarization markers (iNOS, CD86, TNF-α) determined by qPCR. (**G**–**I**) mRNA expression of M2 polarization markers (Arg-1, CD206, IL-10) determined by qPCR. Data are presented as the means ± SEM (N = 3). Statistically significant differences were indicated: * *p* < 0.05, ** *p* < 0.01, *** *p* < 0.001, **** *p* < 0.0001, ns *p* > 0.05, compared to the LPS group.

We evaluated the impact of CFE, luteolin and kaempferol on cell viability and ROS levels in BV2 cells. The CCK-8 assay revealed that CFE significantly attenuated LPS-induced cytotoxicity in a concentration-dependent manner, with the most pronounced improvement in BV2 cell viability observed at 400 μg/mL (*p* < 0.01), prompting the selection of this concentration for further experiments (Figure 8A). Both luteolin (14 μM) and kaempferol (10 μM) also enhanced BV2 cells viability (*p* < 0.001, Figure 8B). Moreover, CFE, luteolin, and kaempferol significantly suppressed ROS generation (Figure 8C, *p* < 0.05, *p* < 0.01, and *p* < 0.01, respectively). The findings indicated that CFE, Luteolin, and Kaempferol confer protection against inflammatory and oxidative stress in BV2 cells.

### 3.14. CFE, Luteolin and Kaempferol Suppressed M1 Polarization and Promoted M2 Polarization in BV2 Microglial Cells

RT-qPCR analysis showed that CFE, luteolin and kaempferol significantly suppressed M1 marker expression (Figure 8D–F) while elevating M2 marker expression (Figure 8G–I). Specifically, CFE downregulated iNOS, CD86, and TNF-α (all *p* < 0.05) while concurrently upregulating Arg-1, CD206, and IL-10 (all *p* < 0.05). Luteolin significantly decreased iNOS (*p* < 0.01), CD86 (*p* < 0.001), and TNF-α (*p* < 0.01), and increased Arg-1 (*p* < 0.01), CD206 (*p* < 0.001), and IL-10 (*p* < 0.01). Kaempferol downregulated iNOS (*p* < 0.01), CD86 (*p* < 0.001), and TNF-α (*p* < 0.01), and upregulated Arg-1 (*p* < 0.01), CD206 (*p* < 0.001), and IL-10 (*p* < 0.001). Compared to the CFE group, the Luteolin and Kaempferol groups demonstrated superior regulatory effects on M1/M2 polarization of BV2 microglial cells. The results showed that the CFE, Luteolin and Kaempferol attenuated macrophage inflammatory responses in BV2 macrophages by suppressing M1 polarization and promoting M2 polarization.

## 4. Discussion

The rising incidence of cognitive decline, which increasingly affects younger demographics and severely compromises quality of life, identifies HNF as a central driver in its pathogenesis and progression [46,47,48]. However, the development of therapeutics specifically targeting HNF remains limited. In this context, the growing recognition of plant-derived natural products as potential treatments for neurological disorders has opened a new avenue for HNF intervention [26,49]. Our study demonstrated that CFE confers significant protection against cognitive decline in a mouse model of HNF. This protective efficacy is underpinned by a multi-faceted mechanism involving direct anti-neuroinflammatory action, remodeling of the gut microbiota and associated metabolites, modulation of microglial polarization, and subsequent activation of the BDNF/5-HT pathway. Phytochemical analysis further revealed that CFE possesses a rich flavonoid profile, with luteolin and kaempferol identified as the predominant constituents, which are considered primary contributors to its observed therapeutic effects.

Our initial experiments used in vitro models to assess the neuroprotective and antioxidant potential of CFE. Pretreatment with CFE demonstrated substantial efficacy in protecting HT22 cells against HNF, with the solvent C extract (chloroform-methanol, 6:4, *v*/*v*) exhibiting the strongest activity and therefore being selected for subsequent investigation. The marked recovery in cell viability and pronounced reduction in ROS levels confirmed that CFE confers protection against the intertwined pathologies of inflammatory and oxidative stress in HNF [50]. To identify the active constituents responsible for these effects, we characterized the chemical composition of CFE. HPLC-MS analysis revealed a complex profile with multiple identified compounds. Based on their established bioavailability, BBB permeability, and documented anti-inflammatory and neuroimmunomodulatory properties [51], flavonoids were prioritized as the likely active fraction. Among these, luteolin and kaempferol were the most abundant, quantified at concentrations of 10,796.37 ng/mL and 3082.77 ng/mL, respectively.

The neuroprotective potential of CFE observed in cellular models was further validated in a mouse model of LPS-induced HNF and cognitive decline. As reported in previous studies [52], HNF mice in our model exhibited significant cognitive impairment. Following CFE intervention, behavioral performance improved notably: in the Morris water maze test, increased platform crossings, longer time spent, and greater distance traveled in the target quadrant indicated enhanced spatial learning and memory. In the novel object recognition test, an elevated recognition index reflected improved exploratory behavior and discrimination of novel objects. These behavioral benefits were accompanied by amelioration of hippocampal histopathology. CFE intervention effectively reversed key LPS-induced structural alterations in the CA1 region, including disorganized neuronal arrangement, widened intercellular spaces, nucleolar condensation, and significant reductions in neuronal density and eosinophilic proportion. Together, these histopathological findings demonstrate that CFE effectively preserves hippocampal structural integrity under neuroinflammatory conditions.

As key pathological drivers in HNF, both the accumulation of Aβ1–42 and the aberrant hyperphosphorylation of Tau protein are directly promoted by the inflammatory process. Consequently, strategies aimed at inhibiting Aβ1–42 accumulation and P-Tau formation, while mitigating their neurotoxicity, are crucial for preventing and treating HNF [5]. Accumulating evidence suggests that certain plant-derived natural products and their bioactive components can counteract HNF by suppressing Aβ1–42 aggregation and Tau hyperphosphorylation. For instance, *Scrophularia buergeriana* extract was shown to reduce Aβ1–42 deposition and Tau hyperphosphorylation in a mouse model of cognitive decline [53]. In line with this, our data from IHC and Western blot analyses demonstrated that CFE significantly suppressed the expression of Aβ1–42, the kinase GSK-3β, total Tau, and Tau phosphorylated at Ser404. GSK-3β is a pivotal kinase that, upon activation, potently drives Tau phosphorylation, leading to neurofibrillary tangle formation and subsequent neuronal dysfunction [7]. Our results suggest that CFE reduces the expression of Aβ1–42 and GSK-3β, thereby inhibiting p-Tau (Ser404) formation by blocking Aβ1–42-mediated aggregation. This mechanism likely contributes to the protection of neuronal and synaptic structure, ultimately improving HNF and cognitive decline, which is consistent with previous research [54].

Synaptic plasticity, which underlies learning and memory, depends critically on neurotrophic support and the maintenance of neuronal structural integrity; thus, enhancing synaptic function represents a promising therapeutic strategy for HNF [55]. BDNF acts as a master regulator of neuronal survival, differentiation, and synaptic function, and its expression in the hippocampus is particularly vital for cognitive processes [56]. Similarly, MAP2 is essential for dendritic arborization and stability, which are fundamental for synaptic connectivity and information integration [57]. Previous studies have shown that luteolin significantly restored the BDNF-TrkB signaling axis and increased MAP2 protein levels, thereby attenuating HNF in mice [58]. In the present study, we found that CFE significantly upregulated hippocampal levels of both BDNF and MAP2, indicating its role in promoting a neuroplastic milieu. This enhancement of neurotrophic signaling and structural support likely represents a key mechanism through which CFE facilitates the recovery of cognitive function, as observed in our behavioral tests.

The neuroprotective and synaptoprotective effects of CFE were accompanied by a marked suppression of HNF. Neuroinflammatory responses are typically driven by aberrant activation of glial cells [59]. Pathological activation of microglia and astrocytes triggers the release of pro-inflammatory cytokines, ultimately contributing to neuronal damage and synaptic loss [14]. In our study, CFE significantly reduced both the protein and mRNA levels of key pro-inflammatory cytokines in the hippocampus and in systemic circulation. Furthermore, CFE markedly downregulated hippocampal expression of the surface receptor CD11b, a marker of microglial activation that is upregulated during HNF and contributes to pro-inflammatory signaling [4]. Immunofluorescence analysis further demonstrated that CFE reduced the activation of both microglia (Iba1+) and astrocytes (GFAP+), indicating that CFE exerts an anti-inflammatory effect on hippocampal glial cells.

The microbiota-gut-brain axis functions as a key bidirectional communication network, linking the gut microbiota with the central nervous system and maintaining a dynamic equilibrium among the gut, brain, and microbial communities [60]. Homeostasis of the gut microbiota, characterized by balanced composition and diversity, is essential for overall health [61]. Studies on the therapeutic application of natural products have shown that plant-derived floral extracts can modulate intestinal microecology and exert prebiotic effects. For example, flavonoids from *Dendrobium officinale* flowers alleviated cognitive decline by reshaping the gut microbiota and suppressing microglial activation, which in turn upregulated BDNF and synaptic proteins and improved synaptic plasticity [49]. In the present study, 16S rRNA sequencing revealed that CFE effectively counteracted LPS-induced gut dysbiosis. It significantly increased the abundance of beneficial bacterial families with known SCFAs-producing capabilities, such as *Muribaculaceae* and *Lachnospiraceae*, while suppressing potential pathobionts like *Staphylococcus* and *Helicobacter*. These microbial changes are functionally important: *Muribaculaceae* and *Lachnospiraceae* are consistently associated with anti-inflammatory activity, gut barrier integrity, and SCFAs production [62,63], whereas *Staphylococcus* and *Helicobacter* have been positively correlated with systemic inflammation, increased gut permeability, and risk for hippocampal neuroinflammation and cognitive decline [64,65]. These results confirm that CFE can restore intestinal microbial health by modulating gut flora structure. The regulatory influence of gut microbiota metabolites on the gut-brain axis is largely mediated by SCFAs. Produced through microbial fermentation of dietary fiber, major SCFAs-acetate, propionate, and butyrate-cross the BBB and perform critical functions such as modulating microglial activity, suppressing HNF, and supporting cognitive processes [66]. Our targeted metabolomics data showed that CFE significantly restored fecal SCFAs levels, with acetate exhibiting a particularly marked increase. This rise in SCFAs provides a mechanistic link between CFE-induced gut microbiota remodeling and the alleviation of brain pathology, likely through microglial modulation and reinforcement of the BBB.

To characterize the systemic metabolic changes induced by CFE, we performed untargeted metabolomic profiling of serum. The analysis indicated that CFE administration altered the host metabolome, upregulating key metabolites involved in glycerophospholipid metabolism, α-linolenic acid (ALA) metabolism, and tryptophan metabolism. ELISA confirmed a central finding: elevated levels of ALA and 5-HT in both serum and hippocampal tissues. ALA, a plant-derived omega-3 fatty acid, exhibits multiple neuroprotective properties, including direct inhibition of Tau aggregation, attenuation of Aβ-induced neurotoxicity, and potent anti-inflammatory and antioxidant activities [67,68]. 5-HT, a key monoamine neurotransmitter, is integral not only to regulating mood, sleep, and cognition but also to modulating neuroimmune responses, as its receptors are expressed on microglia and other immune cells [69]. A Pearson correlation-based network further reinforced the gut-brain link. It showed that the abundances of beneficial bacteria (*Lachnospiraceae*, *Muribaculaceae*) were positively correlated with ALA, 5-HT, and SCFAs, whereas pathogenic genera (*Staphylococcus*, *Helicobacter*) exhibited strong negative correlations. These results suggest that CFE facilitates a coherent gut microbiota-metabolite-brain signaling axis, whereby the restructured gut microbial community enhances the production of beneficial systemic metabolites that can cross the BBB to support brain health and function.

Luteolin treatment has been shown to shift microglial polarization toward the anti-inflammatory M2 phenotype in both in vitro and in vivo studies [17]. Kaempferol, a major flavonoid in edible plants, has been shown to modulate microglial M1/M2 polarization, thereby alleviating neuroinflammatory dyshomeostasis [18]. To determine how the phytochemical profile of CFE influences brain immunity, we examined its direct effects on microglia, the principal cellular mediators of HNF. In BV2 microglial cells, a key finding was that both CFE and its principal flavonoid constituents, luteolin and kaempferol, significantly shifted polarization from the pro-inflammatory M1 phenotype toward the anti-inflammatory M2 state. At the mRNA level, this shift was characterized by downregulation of M1 markers (iNOS, CD86, TNF-α) and upregulation of M2 markers (Arg-1, CD206, IL-10). Notably, luteolin and kaempferol alone induced these changes more potently than the complete CFE extract. These results establish a direct link between the chemical constituents of CFE and the suppression of HNF and glial activation in vitro. We conclude that luteolin and kaempferol mediate the effects of CFE by driving microglial phenotype switching, which in turn disrupts persistent neuroinflammatory signaling and helps establish a microenvironment conducive to neural repair and plasticity.

## 5. Conclusions

In summary, this study demonstrates that CFE is a promising therapeutic candidate for mitigating HNF and cognitive decline. The protective efficacy of CFE is mediated through a multi-level mechanism: it provides direct neuroprotection against oxidative and inflammatory stress; inhibits core neuropathology by reducing Aβ1–42 and P-Tau levels; promotes neuroplasticity via upregulation of BDNF and MAP2; attenuates neuroinflammation by suppressing microglial and astrocyte activation while modulating cytokine networks; remodels the microbiota-gut-brain axis to enhance production of beneficial metabolites such as SCFAs, ALA and 5-HT; and facilitates microglial polarization toward the anti-inflammatory M2 phenotype, an effect likely attributable to its flavonoid constituents, especially luteolin and kaempferol. Collectively, these findings validate the neuroprotective role of CFE via the microbiota-gut-brain axis and provide novel perspectives for preventing and managing cognitive decline with natural compounds. A limitation of the current study lies in its reliance on animal models, which may not fully recapitulate human disease. Thus, these promising preclinical results warrant further validation in clinical trials.

## Figures and Tables

**Table 1:** Metabolites Identified in CFE.

ID.	Identified Compound	Formula	Rt (min)	M/Z
Terpenoids				
1	Valtrate	C_10_H_12_O_5_	6.9529	211.06103219999
2	Loganic acid	C_16_H_24_O_10_	7.1395	377.1417847
3	Geniposide	C_17_H_24_O_10_	7.1689	387.1306856
4	Aucubin	C_15_H_22_O_9_	7.6937	351.1048213
5	Geniposidic acid	C_16_H_22_O_10_	7.1890	355.1044042
6	Verbenalin	C_17_H_24_O_10_	7.8022	387.1307178
7	Asperuloside	C_18_H_22_O_11_	11.5447	435.094409
8	Genipin	C_11_H_14_O_5_	11.7112	209.080969099999
9	Carvone	C_10_H_14_O	13.5809	133.1013854
10	Kahweol	C_20_H_26_O_3_	19.5627	332.2196618000
Flavonoids				
11	Cynaroside	C_21_H_20_O_11_	7.0215	449.1077437
12	Catechin	C_15_H_14_O_6_	7.7130	291.0863623
13	Hesperetin	C_16_H_14_O_6_	8.2874	303.0868847
14	Kaempferol	C_15_H_10_O_6_	8.9881	287.054886
15	Orientin	C_21_H_20_O_11_	9.6627	493.0997219
16	Myricetin	C_15_H_10_O_8_	11.1222	319.0452683
17	Luteolin	C_15_H_10_O_6_	12.8466	285.0411301
18	Fisetin	C_15_H_10_O_6_	14.4979	285.0411181
19	Liquiritigenin	C_15_H_12_O_4_	17.2008	257.0810664
20	Quercetin	C_15_H_10_O_7_	21.5714	303.0499775
Phenylpropanoids				
21	Melilotoside	C_15_H_18_O_8_	7.3099	325.0939562
22	Sinapic acid	C_11_H_12_O_5_	7.5178	225.07579959999
23	Coniferin	C_16_H_22_O_8_	8.4912	341.1246012
24	Clemaphenol A	C_20_H_22_O_6_	8.5585	341.1384034
25	Arctiin	C_27_H_34_O_11_	8.5684	579.2101222
26	Caffeic acid	C_9_H_8_O_4_	8.7172	181.0495659
27	Ferulic acid	C_10_H_10_O_4_	10.4062	193.050046
28	Daphnetin	C_9_H_6_O_4_	6.6184	179.0342196
29	Matairesinol	C_20_H_22_O_6_	12.3796	357.1353564
30	Coumarin	C_9_H_6_O_2_	15.2106	147.0441273
Alkaloids				
31	Aminophylline	C_7_H_8_N_4_O_2_	2.0131	225.0613935
32	Trigonelline	C_7_H_7_NO_2_	2.1357	138.0550452
33	Niacinamide	C_6_H_6_N_2_O	2.1357	123.0554621
34	Nicotinic acid	C_6_H_5_NO_2_	2.2493	124.0395691
35	Picolinic acid	C_6_H_5_NO_2_	3.0782	124.0396142
36	Pipecolic acid	C_6_H_11_NO_2_	3.0782	130.0864943
37	Xanthosine	C_10_H_12_N_4_O_6_	3.4114	283.069145
38	Dopamine	C_8_H_11_NO_2_	9.9235	118.0654293
39	Cepharanthine	C_37_H_38_N_2_O_6_	12.6136	611.2462267
40	Irinotecan	C_33_H_38_N_4_O_6_	26.3161	609.271034799999
Polyketides				
41	Gentiacaulein	C_15_H_12_O_6_	7.2492	287.05699489999
42	Isogentisin	C_14_H_10_O_5_	8.0218	303.0518434
43	Emodin 8-glucoside	C_21_H_20_O_10_	11.8486	455.095751599999
44	Chrysophanein	C_21_H_20_O_9_	12.0973	415.1043764
45	Franguloside	C_21_H_20_O_9_	14.1133	461.1099776
46	Aloe-emodin	C_15_H_10_O_5_	14.2320	269.0461515
47	Rheic acid	C_15_H_8_O_6_	14.4979	283.0254713999
48	Chrysophanic acid	C_15_H_10_O_4_	15.2399	313.0726244
49	Kavain	C_14_H_14_O_3_	16.9331	231.1017323
Shikimate/acetate-malonate pathway derived compounds				
50	Trans-Piceid	C_20_H_22_O_8_	9.6816	411.1068254
51	Yakuchinone-A	C_20_H_24_O_3_	10.8562	347.1407132
52	Batatasin I	C_17_H_16_O_4_	11.2297	343.119330399999
53	Resveratrol	C_14_H_12_O_3_	11.5252	273.0776406999
54	Curcumin	C_21_H_20_O_6_	13.3306	349.1072668
55	6-paradol	C_17_H_26_O_3_	14.1811	279.193054
56	Pinosylvin	C_14_H_12_O_2_	14.8899	213.0909792
57	6-gingerol	C_17_H_26_O_4_	19.0101	295.1906158
58	Gingerol	C_17_H_26_O_4_	22.1175	349.199659
Amino acid related compounds				
59	Linustatin	C_16_H_27_NO_11_	7.8120	464.1763025
60	Amygdalin	C_20_H_27_NO_11_	9.1630	475.194189
61	Glucoerucin	C_12_H_23_NO_9_S_3_	10.3185	439.0846983
62	Triglochinin	C_14_H_17_NO_10_	11.8486	401.1208912
63	Glucoberteroin	C_13_H_24_NO_9_S_3-_	12.1166	415.0458546
64	Lucuminoside	C_19_H_25_NO_10_	12.9959	445.1834607
65	Betanin	C_24_H_26_N_2_O_13_	15.8566	595.1469962
Fatty acids related compounds				
66	Panaxytriol	C_17_H_26_O_3_	17.3533	323.1871353
67	Caprylic acid	C_8_H_16_O_2_	18.5133	287.2234452
68	Gamma-Linolenic acid	C_18_H_30_O_2_	20.0594	323.2235911
69	Jasmonic acid	C_12_H_18_O_3_	20.5980	211.132915
70	Palmitoleic acid	C_16_H_30_O_2_	21.9555	255.2319082
71	Palmitic acid	C_16_H_32_O_2_	22.3487	255.2330876
72	Vaccenic acid	C_18_H_34_O_2_	24.7196	283.26346639999
Others				
73	Pyrogallol	C_6_H_6_O_3_	0.6018	127.03927209999
74	Gallic acid	C_7_H_6_O_5_	1.5681	169.0135405
75	Juglone	C_10_H_6_O_3_	6.9224	175.0392134
76	Ptaquiloside	C_20_H_30_O_8_	7.1493	443.1933204

**Table 2:** Quantification of major identified flavonoids compounds in the CFE.

ID.	Identified Compound	Concentration (ng/mL, Mean ± SD)
1	Luteolin	10,796.3674 ± 65.53
2	Kaempferol	3082.77 ± 110.12
3	Orientin	339.54 ± 22.03
4	Hesperetin	336.42 ± 12.40
5	Liquiritigenin	182.71 ± 31.67
6	Myricetin	170.84 ± 2.74
7	Catechin	65.67 ± 2.60
8	Cynaroside	62.76 ± 8.91
9	Fisetin	15.73 ± 5.60
10	Quercetin	4.60 ± 0.61

## Data Availability

We permit unrestricted use, distribution, and reproduction in any medium, provided the original work is properly cited. Sequencing data generated in this study were deposited in the Sequence Read Archive (SRA) (https://www.ncbi.nlm.nih.gov/sra/, accessed on 19 November 2025) with the BioProject ID: PRJNA1366079 and MTBLS12026.

## Supplementary Materials

The following supporting information can be downloaded at: https://www.mdpi.com/article/10.3390/nu17243958/s1, Table S1: Primer sequence.

## Abbreviations

The following abbreviations are used in this manuscript:

HNF	Hippocampal Neuroinflammation
CFE	*Corylus heterophylla* Fisch. Male Flower Extract
LPS	Lipopolysaccharide
Aβ1–42	Amyloid-beta 1–42
P-Tau	Phosphorylated Tau Protein
GSK-3β	Glycogen Synthase Kinase-3β
IL-33	Interleukin-33
BDNF	Brain-derived Neurotrophic Factor
MAP2	Microtubule-associated Protein 2
ALA	α-linolenic Acid
5-HT	Serotonin
NFTs	Neurofibrillary Tangles
BBB	Blood-Brain Barrier
CD11b	Cluster of Differentiation 11b
ROS	Reactive Oxygen Species
SCFAs	Short-chain Fatty Acids
DMEM	Dulbecco’s Modified Eagle Medium
FBS	Fetal Bovine Serum
P/S	Penicillin-streptomycin
CA1	Cornu Ammonis 1
OTUs	Operational Taxonomic Units
DCFH-DA	2′,7′-dichlorofluorescin Diacetate
