# Supplementary material for: From Microbiota to Metabolomics: How Corylus heterophylla Fisch. Male Flower Extract Shields Mice from Cognitive Decline
Source: Nutrients. 2025 Dec 18;17(24):3958. doi: 10.3390/nu17243958 (PMC12736303; doi:10.3390/nu17243958)
Supplement: Supplementary file 1 [file nutrients-17-03958-s001.zip › nutrients-4025303-supplementary.pdf]

Table S1. Primer sequence

Gene	Primer Sequences
TNF-α	F:ACTACCTCAACCGTTCCA
	R:GAGCTTCCCAGATCACAG
IL-1β	F:AGGAGCACCTCGGTATCA
	R: GTATTGCCATCAGCGTCC
IL-33	F:CTGTTAGTTTTGTTTTGGA
	R:GTAGTAGCACCTGGTCTTG
IL-10	F:TGTGTGTTGGCTGAATTGT
	R:CTGCTCCTGGTGAGTCCTT
iNOS	F:GGACCCAGTGCCCTGCTTT
	R:CACCAAGCTCATGCGGCCT
CD86	F:CTGCACGTCTAAGCAAGGTC
	R:CAG AACACACACAACGGTCA
Arg-1	F:AGTCTGGCAGTTGGAAGC
	R:TGGTTGTCAGGGGAGTGT
CD206	F:ACCCAAGGGCTCTTCTAA
	R:TGGCCTCTTGAGGTATGT
β-actin	F:ATGGTCACGCACGATTTCCC
	R:GAGACCTTCAACACCCCAGC
